# Mucinous Breast Carcinoma: Clinicopathological Comparison With Invasive Ductal Carcinoma

**DOI:** 10.7759/cureus.13650

**Published:** 2021-03-02

**Authors:** Atif A Hashmi, Shamail Zia, Syed Rafay Yaqeen, Omer Ahmed, Ishaq Azeem Asghar, Sabeeh Islam, Anoshia Afzal, Muhammad Irfan, Fazail Zia, Javaria Ali

**Affiliations:** 1 Pathology, Liaquat National Hospital and Medical College, Karachi, PAK; 2 Pathology, Ziauddin University, Karachi, PAK; 3 Internal Medicine, Baqai Medical University, Karachi, PAK; 4 Internal Medicine, Liaquat National Hospital and Medical College, Karachi, PAK; 5 Pathology, Ascension St. John Hospital, Detroit, USA; 6 Internal Medicine, St. Vincent Health Center, Buffalo, USA; 7 Internal Medicine, Faisalabad Medical University, Faisalabad, PAK; 8 Pathology, University of Oklahoma Health Sciences Center, Oklahoma City, USA; 9 Statistics, Liaquat National Hospital and Medical College, Karachi, PAK; 10 Pathology, Jinnah Sindh Medical University, Karachi, PAK

**Keywords:** mucinous carcinoma, invasive ductal carcinoma, estrogen receptor, progesterone receptor, human epidermal growth factor receptor 2

## Abstract

Introduction

Invasive ductal carcinoma (IDC) is the most common histological subtype of breast cancer. Conversely, many special types of breast carcinoma were described with varying prognosis and hormone receptor status. Mucinous carcinoma (MC) is a rare special subtype of breast cancer, and only a few studies have evaluated the clinicopathological and hormone receptor profile of this type of breast cancer. Therefore, in this study, we compared the clinicopathological characteristics of MC with IDC in our population.

Methods

A retrospective observational study was conducted in the Department of Histopathology, Liaquat National Hospital and Medical College, from January 2013 till December 2020, for eight years. During this period, 38 cases of MC were diagnosed and 1268 cases of IDC were identified. All specimens were grossed according to standard protocols and representative sections were submitted from tumors, resection margins, and lymph nodes. Slides were examined by histopathologists to determine tumor type and grade. Immunohistochemical (IHC) stains were applied to evaluate estrogen receptor (ER), progesterone receptor (PR), Ki67, and human epidermal growth factor receptor 2 (HER2/neu) statuses.

Results

The mean age of the patients with MC was 56.47±13.90 years, and most of the patients were above 50 years of age. The mean tumor size was 34.89±19.70 mm. Most tumors were grade 1 (68.4%) with a low mean Ki67 index (15.21±14.06%). Axillary metastasis was present in 31.6% of cases and all of them were nodal (N)-stage N1. ER, PR, and HER2/neu positivity were noted in 94.7%, 78.9, and 10.5% cases, respectively. Compared with IDC, a significant association of MC was noted with age, Ki67 index, tumor (T)-stage, N-stage, and tumor grade. MC cases had a higher mean age than IDC cases. Comparative analysis revealed that MC had a lower frequency of axillary metastasis, a lower mean Ki67 index, and a lower tumor grade than IDC. About biomarker status, MC was noted to have a higher frequency of ER and PR expression, and a lower frequency of HER2/neu expression than IDC.

Conclusion

MC is a rare subtype of breast cancer. However, it is important to recognize this subtype of breast cancer as it is associated with a prognostically better pathological profile, such as lower tumor grade and Ki67 index, lower frequency of axillary metastasis, higher expression of ER and PR, and lower expression of HER2/neu.

## Introduction

Breast cancer is one of the most common cancers in women and the spectrum of breast cancer is increasing with time [1,2]. The World Health Organization (WHO) 2019 update on the classification of breast tumors described more than ten subtypes of breast cancers. Mucinous carcinoma (MC) can occur at many anatomic sites of the body, for instance, GI, pancreatobiliary and urinary tracts, and lung. However, the prognosis of MC differs at different anatomic locations. Although in GI and pancreatobiliary tracts, mucinous differentiation confers a poor prognosis; alternatively, in the breast, pure MC portends a better prognosis compared to invasive ductal carcinoma (IDC), which is the most common breast cancer [3]. IDC is also known as invasive breast carcinoma of no special type. Conversely, many special types of breast carcinoma were described with varying prognosis and hormone receptor status [4]. A designation of pure special type breast carcinoma is made when more than 90% of the sampled tumor is composed of special type breast carcinoma. Conversely, a tumor that is composed of 50-90% special type carcinoma is termed as mixed carcinoma (a mixture of IDC and special type breast carcinoma). Hormone receptors and human epidermal growth factor receptor-2 (HER2/neu) status play an important role in human breast cancer prognosis and management [5]. Different special type breast cancers display varying proportions of expression of estrogen receptor (ER), progesterone receptor (PR), and HER2/neu. MC is rare and only a few studies have evaluated the clinicopathological and hormone receptor profile of this special type of breast cancer. Therefore, in this study, we compared the clinicopathological characteristics of MC with IDC in our population.

## Materials and methods

**Figure 1:**
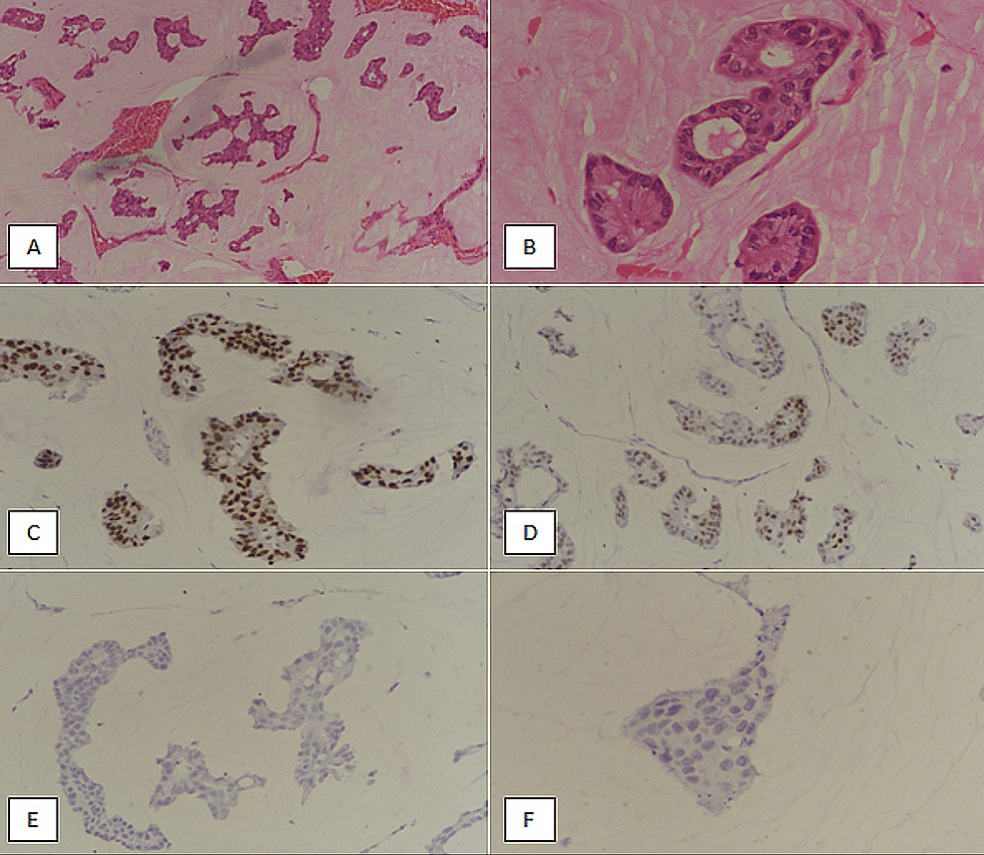
Mucinous breast carcinoma. (A) H & E-staining at 100× magnification showing nests and cluster of tumor cells floating in pools of extracellular mucin. (B) H & E-stained section at 400× magnification showing tumor cells with mild nuclear atypia. (C) ER-staining (IHC) showing diffuse strong expression in 95% of tumor cells. (D) PR-staining (IHC) depicting a strong nuclear expression in 70% of tumor cells. (E) HER2/neu IHC-staining revealing lack of any membranous positivity. (F) Ki67 IHC-staining showing lack of expression in tumor cells. H& E, hematoxylin and eosin; ER, estrogen receptor; IHC, immunohistochemical; PR, progesterone receptor; HER2/neu, human epidermal growth factor receptor 2

A retrospective observational study was conducted in the Department of Histopathology, Liaquat National Hospital and Medical College, from January 2013 till December 2020, for eight years. During this period, 38 cases of MC were diagnosed and 1268 cases of IDC were identified. Only cases with a diagnosis of pure MC (greater than 90% mucinous histology) were included in the study. The included specimens were wide local excisions, simple mastectomies with sentinel lymph node (SLN) dissection, and modified radical mastectomies (MRMs). SLN biopsy was conducted intra-operatively to evaluate lymph node status for clinically and radiologically negative axillary lymph nodes. For patients undergoing mastectomy, any positive SLN with macrometastasis (>2 mm) was followed by axillary dissection. Alternatively, for patients undergoing breast conservation surgery, three or more positive SLNs on frozen were followed by axillary dissection. Cases with neo-adjuvant chemotherapy or radiation before surgery were excluded from the study. All specimens were grossed according to standard protocols and representative sections were submitted from tumors, resection margins, and lymph nodes. Slides were examined by histopathologists to determine tumor type and grade. Diagnosis of MC was rendered based on histological features, i.e, nests and clusters of tumor cells floating in pools of extracellular mucin. Tumors with more than 90% mucinous histology were labeled as MC. Immunohistochemical (IHC) stains were applied to evaluate ER, PR, Ki67, and HER2/neu status (Figure 1).

More than 1% nuclear expression of ER and PR in tumor cells was taken as positive. For HER2/neu, strong and complete membranous expression in more than 10% invasive tumor cells was labeled as positive (3+) expression. For cases with equivocal (2+) IHC results for HER/2neu, fluorescence in situ hybridization (FISH) studies were done to evaluate HER2/neu gene amplification. Cases with equivocal (2+) IHC and amplified FISH results were considered HER2/neu positive. Ki67 was interpreted in areas of the tumor with the highest nuclear labeling (hot spots). An average percentage of Ki67 was taken after calculating 500 tumor cells in hot spots.

Data analysis was performed using Statistical Package for Social Sciences (Version 26.0, IBM Inc., Armonk, USA). Independent t-test, chi-square, and Fisher’s exact tests were used to check the association. P-values < 0.05 were considered as significant.

## Results

The mean age of the patients with MC was 56.47±13.90 years, and most of the patients were above 50 years of age. The mean tumor size was 34.89±19.70 mm. Most tumors were grade 1 (68.4%) with a low mean Ki67 index (15.21±14.06%). Axillary metastasis was present in 31.6% of cases and all of them were nodal (N)-stage N1. ER, PR, and HER2/neu positivity were noted in 94.7%, 78.9, and 10.5% cases, respectively (Table 1).

**Table 1:** Clinicopathologic characteristics of mucinous breast carcinoma (n=38) SD, standard deviation; T, tumor; N, nodal; ER, estrogen receptor; PR, progesterone receptor; HER2/neu, human epidermal growth factor receptor 2

Clinicopathologic characteristics and immunohistochemical expression	Values
Age (years), mean±SD	56.47±13.90
Age groups	
<30 years, n (%)	2 (5.3)
31-50 years, n (%)	10 (26.3)
51-70 years, n (%)	24 (63.2)
>70 years, n (%)	2 (5.3)
Ki67 index (%), mean±SD	15.21±14.06
Ki67 index groups	
<15%, n (%)	26 (68.4)
15-24%, n (%)	6 (15.8)
25-44 %, n (%)	4 (10.5)
>44%, n (%)	2 (5.3)
Tumor size (mm), mean±SD	34.89±19.70
T-stage	
T1, n (%)	8 (21.1)
T2, n (%)	20 (52.6)
T3, n (%)	10 (26.3)
Axillary metastasis	
Present, n (%)	12 (31.6)
Absent, n (%)	26 (68.4)
N-stage	
N0, n (%)	26 (68.4)
N1, n (%)	12 (31.6)
Tumor grade	
Grade I, n (%)	26 (68.4)
Grade II, n (%)	6 (15.8)
Grade III, n (%)	6 (15.8)
Laterality	
Left, n (%)	22 (57.9)
Right, n (%)	16 (42.1)
Surgery type	
Modified radical mastectomy, n (%)	16 (42.1)
Simple mastectomy with sentinel lymph node dissection, n (%)	8 (21.1)
Wide local excision with/without axillary dissection, n (%)	14 (36.8)
ER	
Positive, n (%)	36 (94.7)
Negative, n (%)	2 (5.3)
PR	
Positive, n (%)	30 (78.9)
Negative, n (%)	8 (21.1)
HER2/neu	
Positive, n (%)	4 (10.5)
Negative, n (%)	34 (89.5)
Lymphovascular invasion	
Present, n (%)	6 (15.8)
Absent, n (%)	32 (84.2)
Dermal lymphatic invasion	
Present, n (%)	4 (10.5)
Absent, n (%)	34 (89.5)

Table 2 compares the clinicopathological characteristics of MC and IDC. A significant association of MC was noted with age, Ki67 index, tumor (T)-stage, N-stage, and tumor grade. MC cases had a higher mean age than IDC cases. Comparative analysis revealed that MC had a lower frequency of axillary metastasis, a lower mean Ki67 index, and a lower tumor grade than IDC. Conversely, MC had a higher frequency of T1 and T3 stages than T2 (compared to IDC). About biomarker status, MC was noted to have a higher frequency of ER and PR expression, and a lower frequency of Her2/neu expression than IDC. Alternatively, no significant association was noted with tumor size, laterality, lymphovascular invasion, and dermal lymphatic invasion (Table 2).

**Table 2:** Comparison of clinicopathologic characteristics of mucinous carcinoma with invasive ductal carcinoma of the breast. *Independent t-test was applied. **Chi-square test was applied. ***Fisher’s exact test was applied. ****p-Value significant as <0.05. SD, standard deviation; T, tumor; N, nodal; ER, estrogen receptor; PR, progesterone receptor; HER2/neu, human epidermal growth factor receptor 2

Clinicopathological characteristics and immunohistochemical expression	Values		p-Value
	Invasive ductal carcinoma (n=1268)	Mucinous carcinoma (n=38)	
Age (years)*, mean±SD	51.95±12.15	56.47±13.90	0.024****
Ki67 index (%)*, mean±SD	30.54±21.60	15.21±14.06	<0.0001****
Ki67 index groups**			
<15 %, n (%)	362 (28.5)	26 (68.4)	<0.0001****
15-24%, n (%)	286 (22.6)	6 (15.8)	
25-44%, n (%)	282 (22.2)	4 (10.5)	
>44%, n (%)	338 (26.7)	2 (5.3)	
Tumor size (mm)*, mean±SD	36.11±14.84	34.89±19.70	0.708
T-stage**			
T1, n (%)	166 (13.1)	8 (21.1)	0.042****
T2, n (%)	906 (71.5)	20 (52.6)	
T3, n (%)	196 (15.5)	10 (26.3)	
Axillary metastasis**			
Present, n (%)	636 (50.2)	12 (31.6)	0.024****
Absent, n (%)	632 (49.8)	26 (68.4)	
N-stage***			
N0, n (%)	640 (50.5)	26 (68.4)	<0.0001****
N1, n (%)	260 (20.5)	12 (31.6)	
N2, n (%)	170 (13.4)	0 (0)	
N3, n (%)	198( 15.6)	0 (0)	
Tumor grade***			
Grade I, n (%)	106 (8.4)	26 (68.4)	<0.0001****
Grade II, n (%)	586 (46.2)	6 (15.8)	
Grade III, n (%)	576 (45.4)	6 (15.8)	
Laterality**			
Left, n (%)	630 (49.7)	22 (57.9)	0.319
Right, n (%)	638 (50.3)	16 (42.1)	
ER**			
Positive, n (%)	798 (62.9)	36 (94.7)	<0.0001****
Negative, n (%)	470 (37.1)	2 (5.3)	
PR**			
Positive, n (%)	646 (50.9)	30 (78.9)	0.001****
Negative, n (%)	622 (49.1)	8 (21.1)	
HER2/neu**			
Positive, n (%)	446 (35.2)	4 (10.5)	0.002****
Negative, n (%)	822 (64.8)	34 (89.5)	
Lymphovascular invasion**			
Present, n (%)	314 (24.8)	6 (15.8)	0.205
Absent, n (%)	954 (75.2)	32 (84.2)	
Dermal lymphatic invasion**			
Present, n (%)	156 (12.3)	4 (10.5)	1.000
Absent, n (%)	1112 (87.7)	34 (89.5)	

## Discussion

In this study, we found that the frequency of MC is as low as only 38 cases of MC were identified compared to 1268 cases of IDC in the same study period. However, MC was noted to have better prognostic characteristics, such as lower tumor grade and proliferation index, and lower N-stage. Moreover, MC showed a prognostically better biomarker profile, i.e., higher expression of hormone receptors and lower expression of HER2/neu.

MC accounts for approximately 4% of invasive breast carcinomas and is divided into pure and mixed subtypes and mostly occurs in postmenopausal and perimenopausal women [6,7]. It has better overall survival than IDC and invasive lobular carcinoma and the 10-year survival rate is close to 90.4% [8]. Like IDC, MC metastasize to axillary lymph nodes, and therefore determining the nodal status is the most important factor in the management of MC [6,8]. In our study, axillary metastasis was noted in 31.6% of cases of MC. In contrast, axillary metastasis was seen in 50.2% of cases of IDC.

Di Saverio et al. reported the metastatic disease rate of MC to be around 12-14% [9]. The most important prognostic factor for MC is the lymph node status and nodal metastasis indicates a poor prognosis. Skotnicki et al. found that the two subtypes had different nodal status and patients with pure MC had a lower incidence of axillary nodal metastasis than patients with mixed (mucinous and ductal/lobular) carcinomas [10]. The survival rate was also significantly higher in patients with pure mucinous subtypes than those with mixed subtypes [9-11].

Lei et al. found that the lymph node status, tumor size, clinical stage, and p53 mutation rate differed between mixed and pure subtypes and reported that more than 30 mm size of the tumor, p53 mutation, and low mucinous component were significant risk factors for lymph node metastasis in MC patients [11]. Cao et al. retrospectively compared MC and IDC and had similar findings of better overall survival, smaller tumor size, a lower rate of nodal metastasis, the lower stage of the disease, and favorable hormonal profile in MC compared with IDC [12].

The main differential diagnosis of MC is metastasis from the GI tract. However, the breast is an uncommon site for metastasis. In problematic cases, IHC positivity for ER, PR, and mammaglobin in primary MC of breast and CDX2 positivity in metastatic MC from GI tract is helpful. Another differential diagnosis includes matrix-producing carcinoma (a sub-type of metaplastic breast carcinoma). However, matrix-producing carcinoma is frequently high-grade, ER/PR negative, and p63 positive, unlike MC.

We acknowledge that our study had a few limitations, as the number of cases of MC was low. Moreover, follow-up of the patients was not available to compare the difference in overall survival and disease-free survival between MC and IDC cases.

## Conclusions

MC is a distinctive rare subtype of breast carcinoma with overall better prognostic pathological characteristics than IDC. In this study, we noted that MC was associated with lower grade and lower mean Ki67 index than IDC. Similarly, there was a lower frequency of axillary metastasis in MC than IDC. Moreover, a higher frequency of ER and PR expression, and lower frequency of HER2/neu expression portends a better biomarker profile of MC and predicts a better response to hormonal therapy. However, large-scale follow-up studies are advised to compare the cancer-specific survival difference between MC and IDC.
